# Supplementary material for: The role of lattice dynamics in ferroelectric switching
Source: Nat Commun. 2022 Mar 2;13:1110. doi: 10.1038/s41467-022-28622-z (PMC8891289; doi:10.1038/s41467-022-28622-z)
Supplement: Supplementary file 1 — Supplementary Information for the manuscript [file 41467_2022_28622_MOESM1_ESM.pdf]

Supplementary Information for “The role of lattice dynamics in ferroelectric switching”

Section 1: Landau Potential Coefficients[1]

**Parameter**	**Phase field**	**DFT**	**Unit**
α_1	$4*10^5*(T-1193)$	$-2.98*10^9$	$J*m/C^2$
α_{11}	$3*10^8$	$1.38*10^9$	$J*m^5/C^4$
α_{12}	$1.188*10^8$	$2.06*10^9$	$J*m^5/C^4$
β_1	$6*10^6*(T-1198)$	$-4.78*10^{10}$	$J/m^3/rad^2$
β_{11}	$3.44*10^{10}$	$3.89*10^{11}$	$J/m^3/rad^4$
β_{12}	$6.799*10^{10}$	$5.25*10^{11}$	$J/m^3/rad^4$
λ_{11}	0.288	0.261	$1/rad^2$
λ_{12}	-0.097	-0.125	$1/rad^2$
λ_{44}	0.206	-0.825	$1/rad^2$
t_{11}	$4.532*10^9$	$3.96*10^{10}$	$J*m/(C^2*rad^2)$
t_{12}	$2.266*10^9$	$6.40*10^{10}$	$J*m/(C^2*rad^2)$
t_{44}	$-4.84*10^9$	$-1.16*10^{11}$	$J*m/(C^2*rad^2)$
C_{11}	$2.95*10^{11}$	$3.12*10^{11}$	Pa
C_{12}	$1.18*10^{11}$	$1.24*10^{11}$	Pa
C_{44}	$0.74*10^{11}$	$0.78*10^{11}$	Pa
Q_{11}	0.0603	0.0572	m^4/C^2
Q_{12}	-0.0111	-0.0143	m^4/C^2
Q_{44}	0.0176	0.0703	m^4/C^2

Phase-field simulations are performed at room temperature, T=298K. The relative background dielectric constant $\varepsilon_{11}^b,\ \varepsilon_{22}^b, \varepsilon_{33}^b$ is 50. The gradient energy coefficients are G_{1111}= $2.16*10^{-10}Jm^3/C^2$, G_{1122}= $-2.16*10^{-10}Jm^3/C^2$, G_{1212}= $2.16*10^{-10}Jm^3/C^2$.

The value of L_p and L_θ are set to be 1 in reduced unit. To understand the meaning of reduced unit, we rewrite our evolution equation (take the polarization evolution equation as an example) by moving the kinetic coefficient L from the right-hand side of the equation to the left-hand side,

$$\frac{\partial p_i}{\partial(tL_p)} = -\frac{\delta F_{\mathrm{tot}}}{\delta p_i}$$

Therefore, we can absorb the kinetic coefficient into time, by redefining, t→t*L as the new time parameter. It is in this sense, that we claim "reduced units" for L since we absorbed the unit into the time unit. As such, we report arbitrary units of time in our simulation.

Relationship between Voigt notation and tensor notation:

$\alpha_1 = \alpha_{11}$, $\alpha_{11} = \alpha_{1111}$, $\alpha_{12} = 2\alpha_{1122}$

$\beta_1 = \beta_{11}$, $\beta_{11} = \beta_{1111}$, $\beta_{12} = 2\beta_{1122}$

$\lambda_{11} = \lambda_{1111}$, $\lambda_{12} = \lambda_{1122}$, $\lambda_{44} = 4\lambda_{1212}$

$t_{11} = t_{1111}$, $t_{12} = t_{1122}$, $t_{44} = 2t_{1212}$

$C_{11} = C_{1111}$, $C_{12} = C_{1122}$, $C_{44} = C_{1212}$

$Q_{11} = Q_{1111}$, $Q_{12} = Q_{1122}$, $Q_{44} = 4Q_{1212}$

In order to provide fully *ab initio* description of the switching energy landscape in freestanding and clamped films of $BiFeO_3$, we employ the model described by Eq. 1 and the parameter set extracted using DFT calculations. To obtain this parameter set we apply the following procedure: (i) we construct several structural $BiFeO_3$ polymorphs, optimize their crystal structures using DFT and extract their equilibrium properties (polarization, octahedral tilts, strains and energies); (ii) we derive the analytical expressions for all the model parameters using the conditions $\frac{\partial F}{\partial \varphi_i} = 0$ (where *F* is defined by Eq. 1 and φ_i is the order parameter) as well as the expressions for the energies of the considered polymorphs; (iii) we use these analytical expressions and the DFT values of polarization, octahedral tilts, strains and energies to compute the values of the model parameters.

All DFT calculations are performed using the Vienna Ab initio Simulation Package (VASP) [2]. We employ the generalized gradient approximation (GGA+U) for the exchange-correlation potential in the form of Perdew-Burke-Ernzerhof [3] revised for solids (PBEsol) [4]. For more accurate treatment of Fe *3d* electrons of we apply Hubbard-*U* correction U=4 eV. The calculations are performed using the 40-atom supercell of $BiFeO_3$ (the perovskite unit cell is doubled along *x*, *y* and *z* directions) and 3x3x3 Monkhorst-Pack k-point grid was used for integration over the Brillouin zone corresponding to this supercell. The energy cutoff for the plane wave basis is set to 500 eV. In all simulations we impose the G-AFM order of Fe magnetic moments.

Section 2: Note on Calculating in-plane and out-of-plane components of elastic strain

Since the elastic strain is the total strain minus the eigenstrain, the total strain should be equal to the eigenstrain of the original state $\lambda_{ijkl}\theta_k^{ref}\theta_l^{ref} + Q_{ijkl}p_k^{ref}p_l^{ref}$ $(i, j = 1,2)$, where the superscript "ref" denotes the original state before the switching process. Thus the in-plane elastic strain during switching is $\lambda_{ijkl}\theta_k\theta_l + Q_{ijkl}p_k p_l$-$\lambda_{ijkl}\theta_k^{ref}\theta_l^{ref} + Q_{ijkl}p_k^{ref}p_l^{ref}$ $(i, j = 1,2)$. From the traction free surface conditions, $\sigma_{13} = \sigma_{23} = \sigma_{33} = 0$, we can calculate the out-of-plane components of the elastic strain.

Section 3: Numerical Details

We are using a semi-implicit Fourier spectral method [5] to evolve the time dependent Ginzburg-Landau equation. The whole system is in a periodic condition with air/substrate layer above and below the ferroelectric material in the Z direction. For a film simulation, since the grid points outside the film are either air or substrate, the order parameters are initialized with non-zero values within the film and fixed at 0 outside the film.

The mechanical equilibrium, $\sigma_{ij,j} = 0$, is solved using a Fourier spectral solver, which will give us the stress and strain distributions that are necessary to calculate the elastic driving force for order parameters. The mechanical system is periodic in the X and Y directions. To implement the boundary conditions for clamped and free-standing film along Z direction, we used a superposition method that is explained in details in our previous publication[6].

The electrostatic equilibrium equation, $\varepsilon_0(\varepsilon_{11}^b \frac{\partial^2 \Phi}{\partial x^2} + \varepsilon_{22}^b \frac{\partial^2 \Phi}{\partial y^2} + \varepsilon_{33}^b \frac{\partial^2 \Phi}{\partial z^2}) = \frac{\partial p_1}{\partial x} + \frac{\partial p_2}{\partial y} + \frac{\partial p_3}{\partial z}$ is also solved using a Fourier spectral solver, which will give us the electric field distribution that is needed for the electric driving force to the polarization. The electrostatic system is periodic in the X and Y directions. For both the clamped and free-standing film cases, we are using a short-circuit boundary conditions for the film top and bottom surface/interface along Z direction, which means the electric potential is fixed on the top and bottom. The bottom surface/interface is always grounded (*i.e.*, electric potential equals 0). We change the electric potential on the top surface between negative, zero, and positive, depending on whether we are poling, relaxing or switching the system.

Figure S1. Clamped vs. Membrane Switching Energy Landscapes for BTO and PTO. a. and **b.** show calculated switching energy landscapes for prototypical ferroelectrics BTO and PTO, respectively, showing energy barrier height reductions of ~30% in BTO and ~35% in PTO. Calculations are performed by clamping non-switching order parameters.

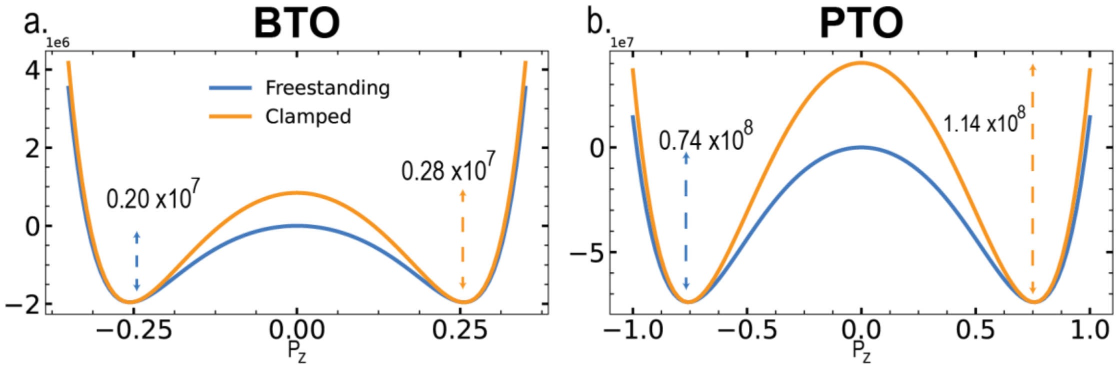

Figure S2. Growth, transfer, and characterization of freestanding BFO membranes. a, Schematic of the SRO/BFO/SRO/LSMO/STO heterostructure growth, freestanding membrane release and transfer. b, AFM images of the BFO film (a) and freestanding membrane (b) with thickness of 35nm. The roughness is 223 pm and 406 pm, respectively.

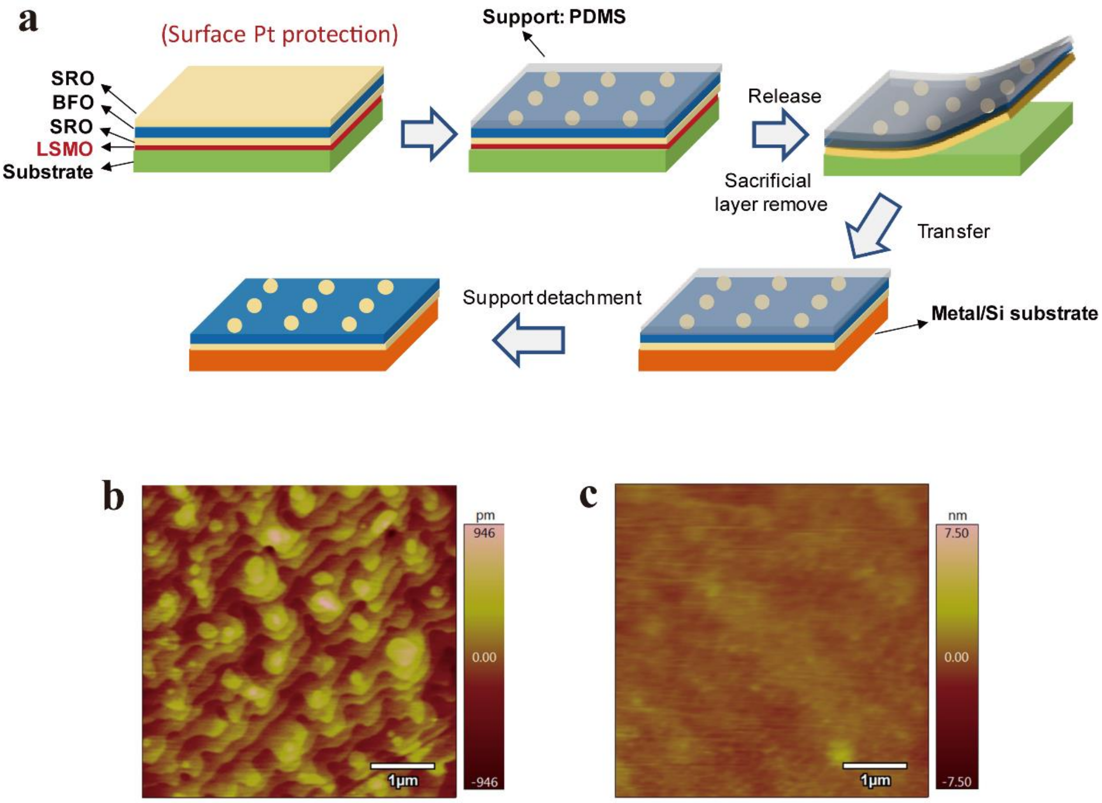

Figure S3. XRD pattern and Reciprocal space maps (RSM) of BFO samples before and after freestanding. a, 2θ-ω XRD patterns of the BFO/SRO/LSMO with BFO thickness of 35 nm before and after lift off. **b, c,** RSM of the sample (b) before and (c) after lift off. **d, e, f,**RSM of the samples after lift off with BFO thickness of 8, 60 and 100 nm, respectively.

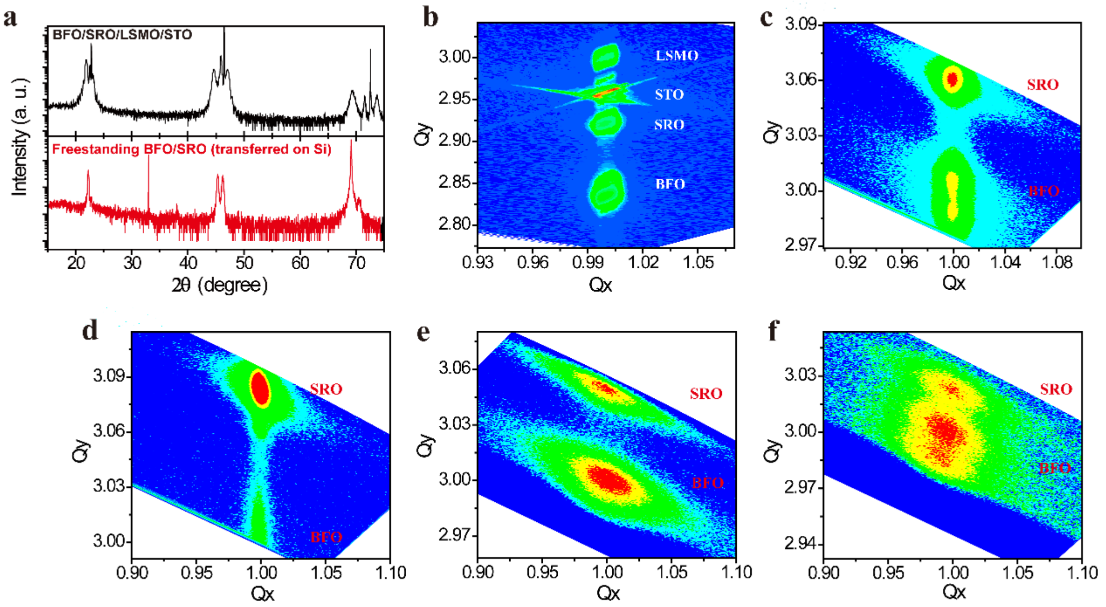

Figure S4. Domain patterns of the 100-nm BFO film and free-standing membrane. a, b, out-plane PFM amplitude images of film (a) and free-standing membrane (b). **c, d**, in-plane PFM phase images of film (c) and free-standing membrane (d). **e, f**, out-plane PFM phase images of film (e) and free-standing membrane (f). The free-standing membrane features larger domain size and an exclusively 180° domain pattern.

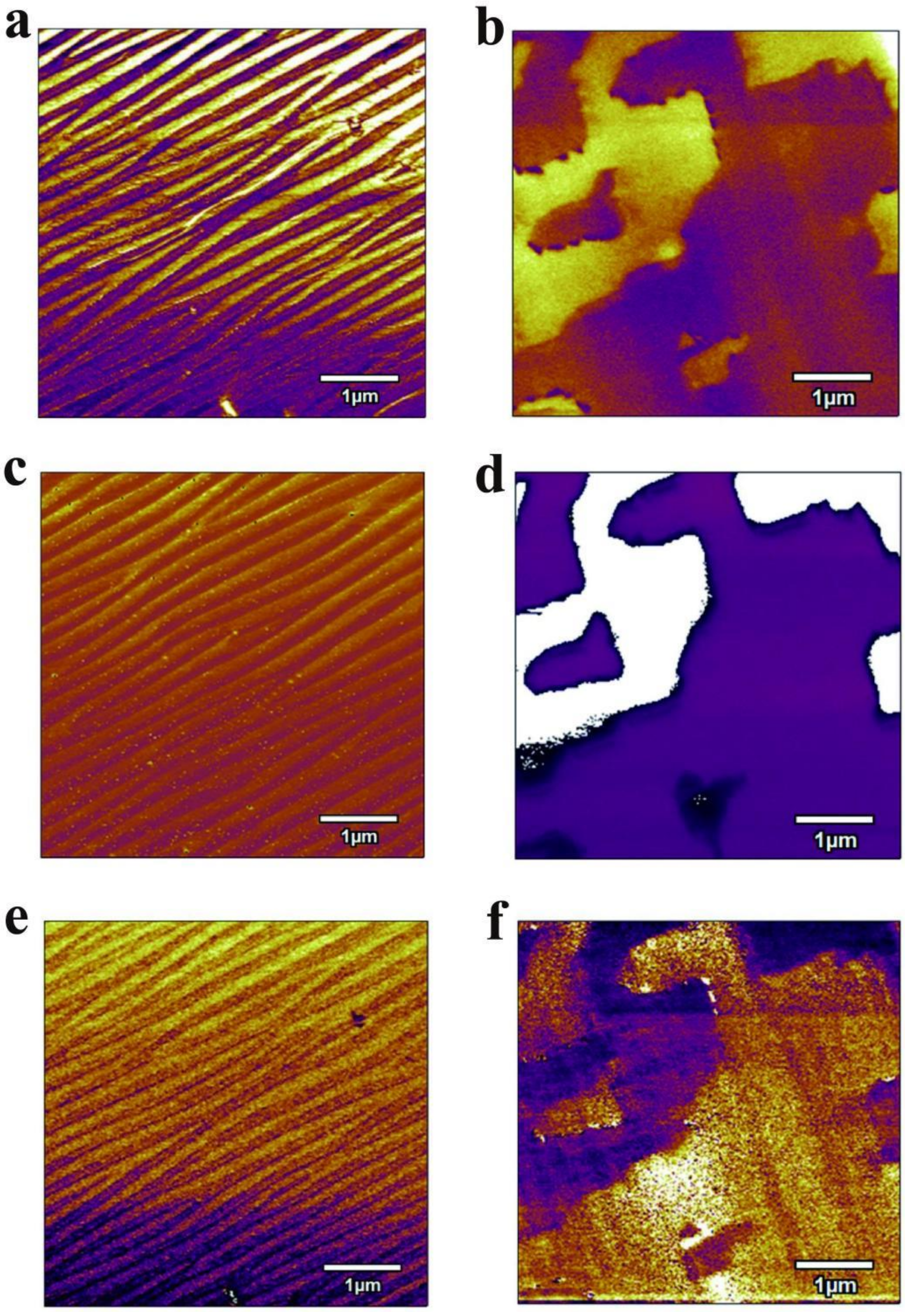

Figure S5. Domain patterns of the BFO films and free-standing membranes with different thicknesses. a, d, in-plane PFM amplitude images of BFO film (a) and free-standing membrane (b) with thickness of 60 nm. **b, e**, in-plane PFM amplitude images of BFO film (b) and free-standing membrane (e) with thickness of 20 nm. **c, f**, in-plane PFM amplitude image of BFO film (c) and free-standing membrane (f) with thickness of 8 nm. All the free-standing membranes feature larger size of domain and the emergence of an exclusively 180° domain pattern.

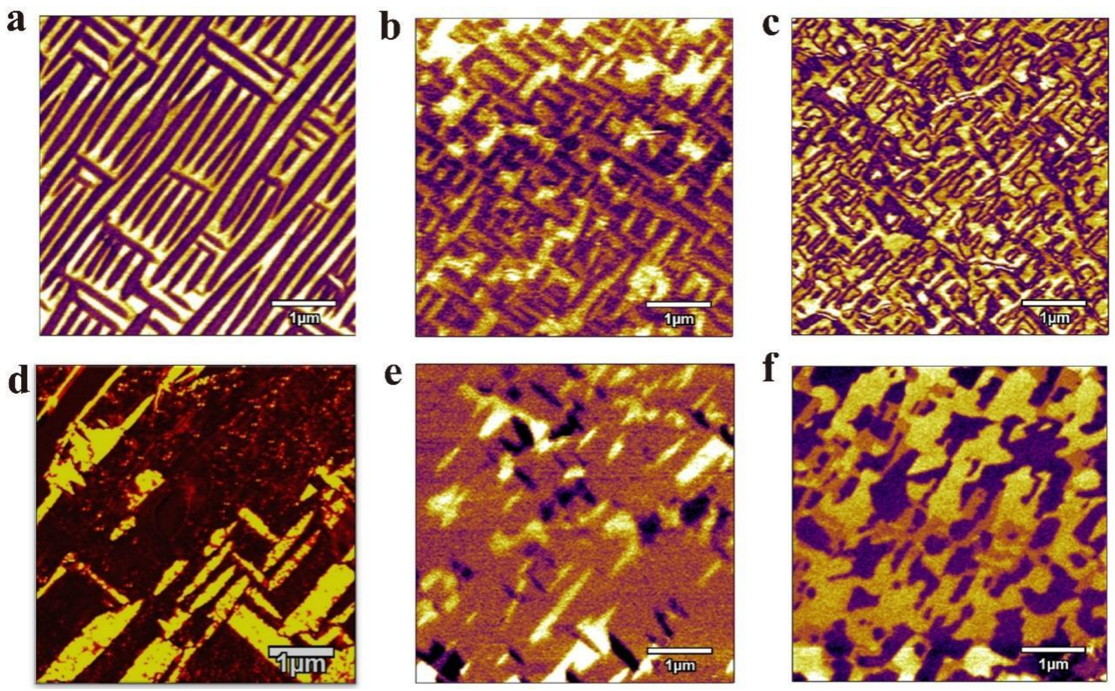

Figure S6. Phase-field simulation of a BFO layer before and after lift-off, with 2-variant initial domain structure. Domain structure of a BFO film with a 2-variant domain walls, before (**a.**, **b.**) and after (**c.**, **d.**) removal of the substrate.

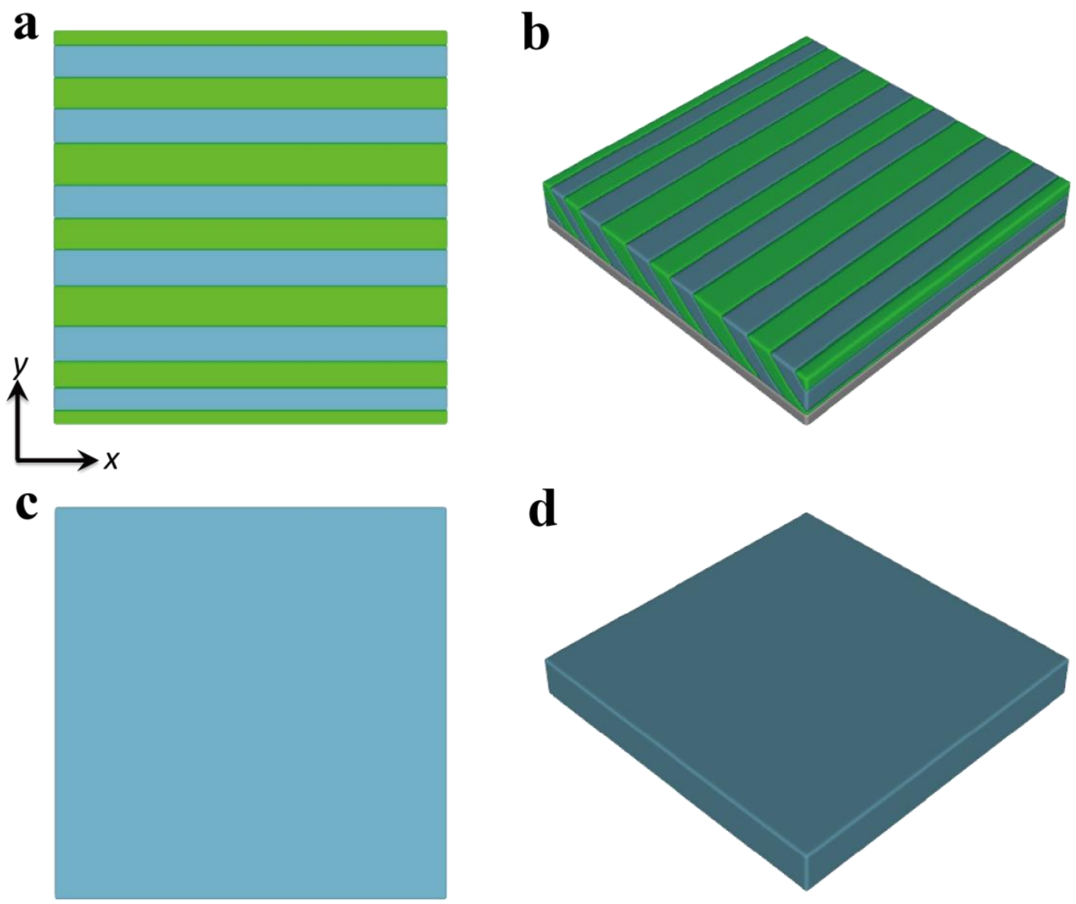

Figure S7. Ferroelectric polarization versus electric voltage (P-V loops) of the BFO films and transferred free-standing membranes. a, b, P-V loops of the SRO/BFO/SRO capacitors for a 100-nm BFO (a) before and (b) after lift-off. The measurements were carried out across a wide frequency range from 1 Hz to 100 kHz. **c, d,** P-V loops of the SRO/BFO/SRO capacitors with 35-nm BFO (c) before and (d) after lift-off. The measurements were carried out across a wide frequency range from 50 Hz to 100 kHz, at temperature of 100 K.

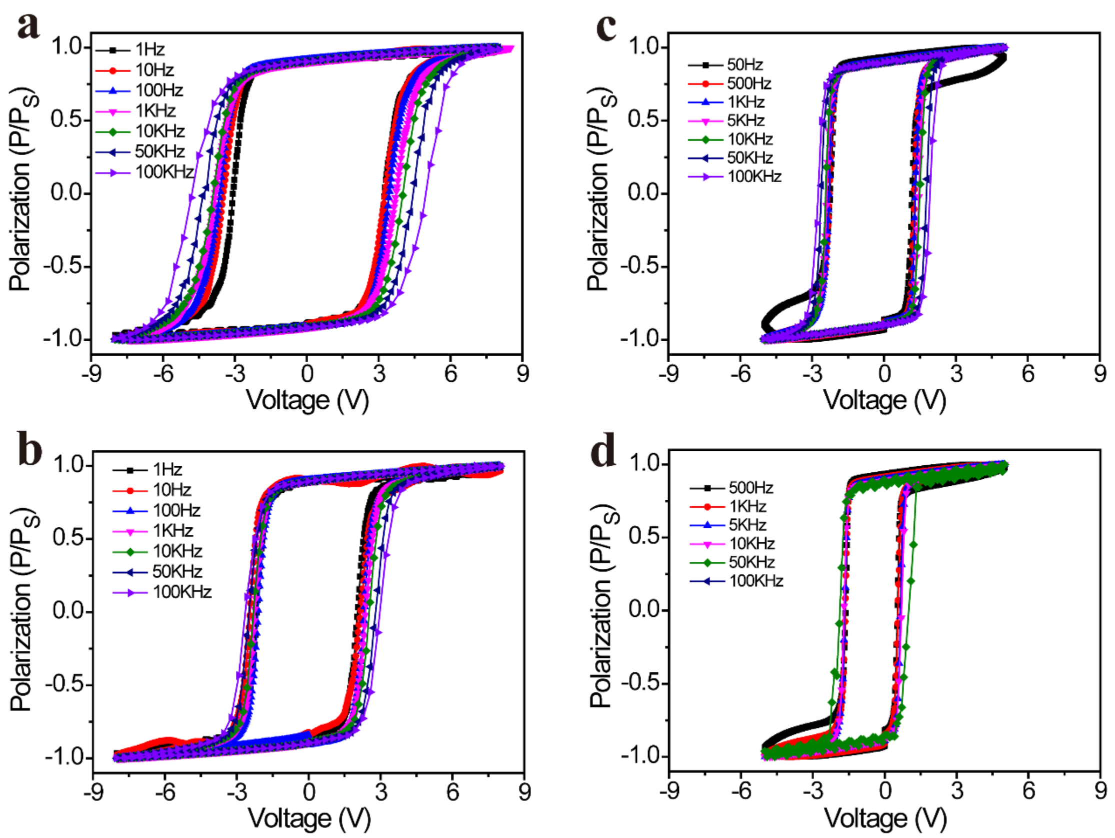

Figure S8. Frequency dispersion of measured Vc. Measured coercive voltage for various excitation frequencies. Fits are discussed in **Table S1.**

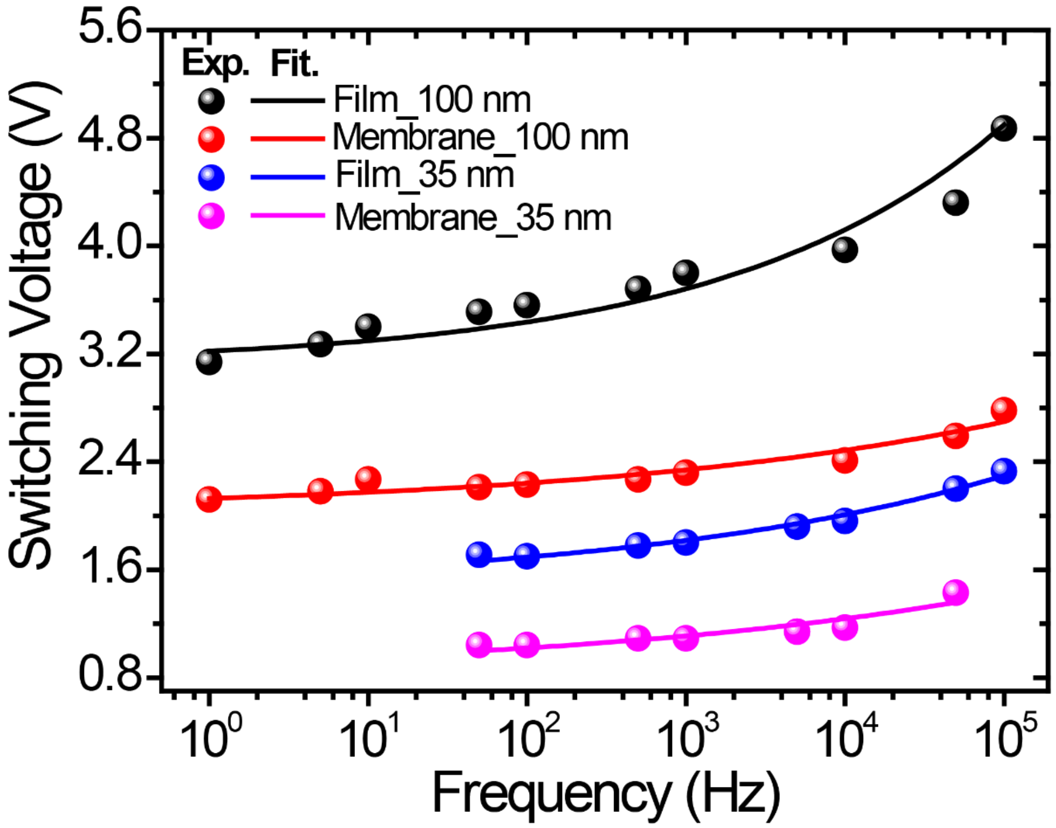

Figure S9. Piezoelectric Phase and Amplitude response of the BFO samples with varied thickness before and after lift-off. a, d, 100 nm. **b, e,** 60 nm, **c, f,** 35 nm. We can see obvious decrease of switching voltage after lift-off.

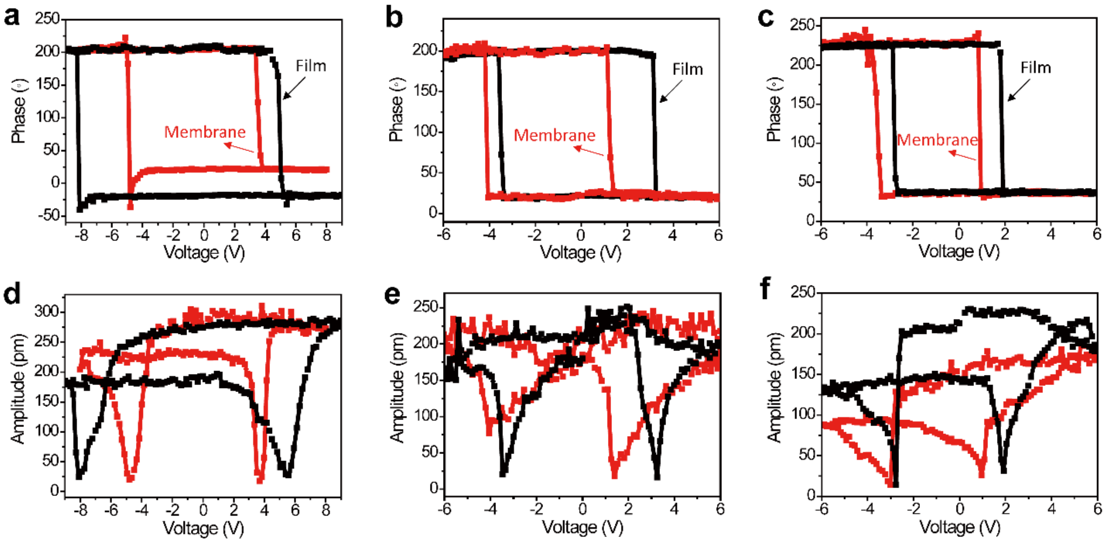

Figure S10. Switching dynamics in the SRO/BFO/SRO capacitors samples before and after lift-off. a, b, The switching current responses for the samples with BFO thickness of 25 nm, before and after lift-off, respectively.

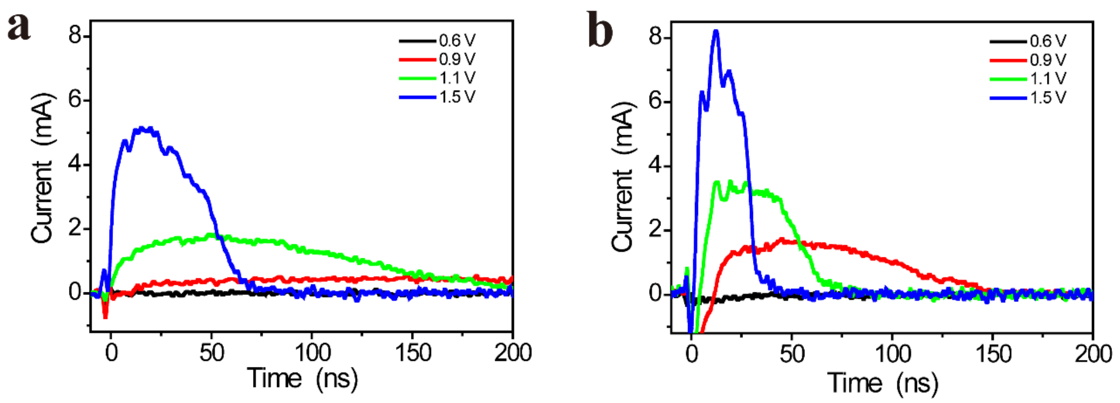

Figure S11. Free energy evolution during the switching process under an externally applied voltage for clamped film and membrane cases without oxygen octahedral tilts. Horizontal axis is the time in arbitrary unit (a.u.) and vertical axis is the average energy of the corresponding component within the whole simulation system in J m^{-3}. **(a)** Elastic energy. **(b)** Electrostatic energy. **(c)** Landau energy. **(d)** Total free energy which is the summation of the elastic electrostatic, and Landau energy.

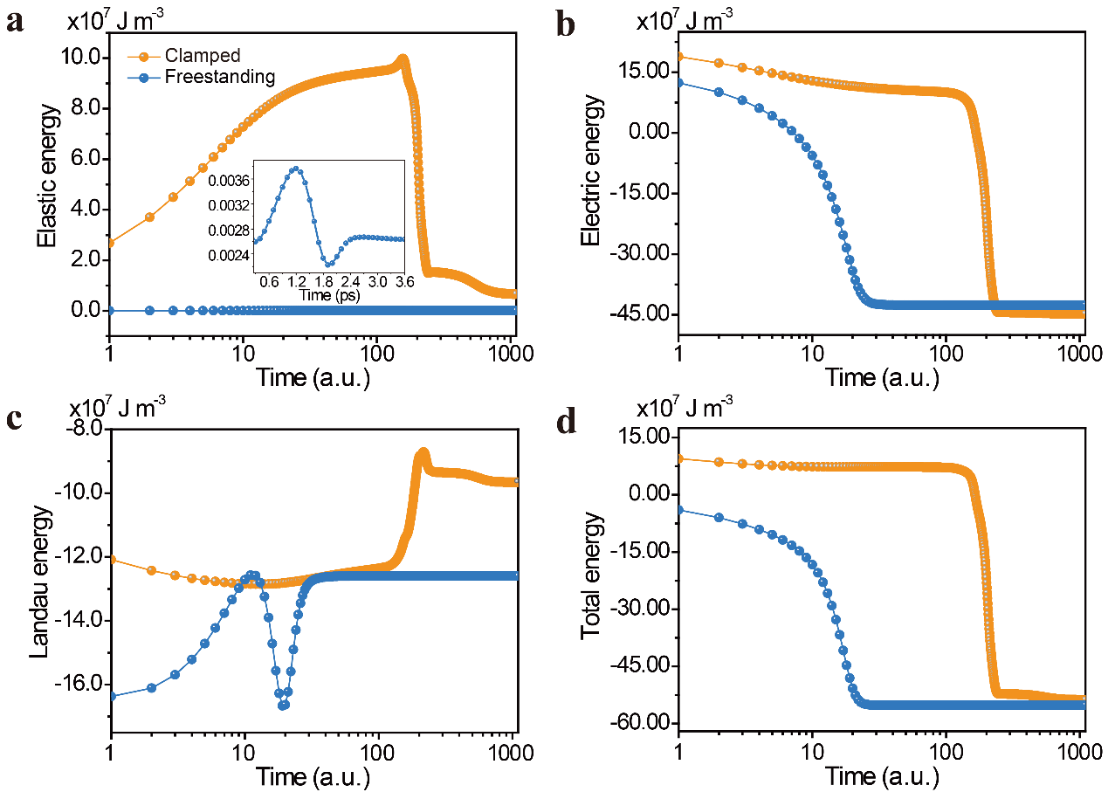

Figure S12. Extended Strain Clamping. Evolution of the polarization, octahedral tilts and strain tensor components during the 109° polarization switching. The curves denoted as "clamped" correspond to the strain clamping case (ϵ_{11}, ϵ_{22} and ϵ_{12} are fixed to their equilibrium values corresponding to the initial direction of the polarization while all the other order parameters are allowed to relax.)

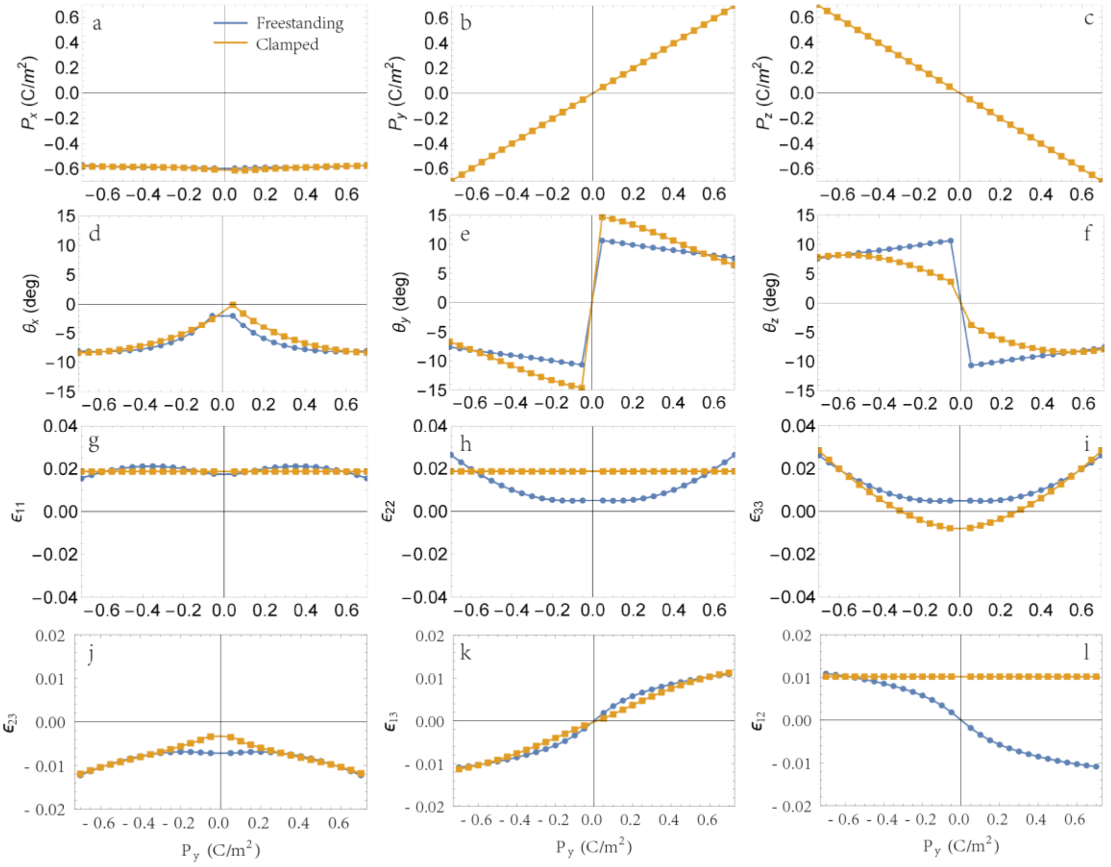

Figure S13 Extended Weak Strain + Tilt Clamping. Evolution of the polarization, octahedral tilts and strain tensor components during the 109° polarization switching. The curves denoted as “clamped” correspond to the “weak strain + tilt clamping” case *(*non-switching components of polarization and tilts (Px and θ_x) as well as $\epsilon_{11,}$ ϵ_{22} and ϵ_{12} components of the strain tensor are fixed to their initial equilibrium values. For each value of P_y only θ_y, θ_z and the unclamped components of strain tensor are allowed to relax*).*

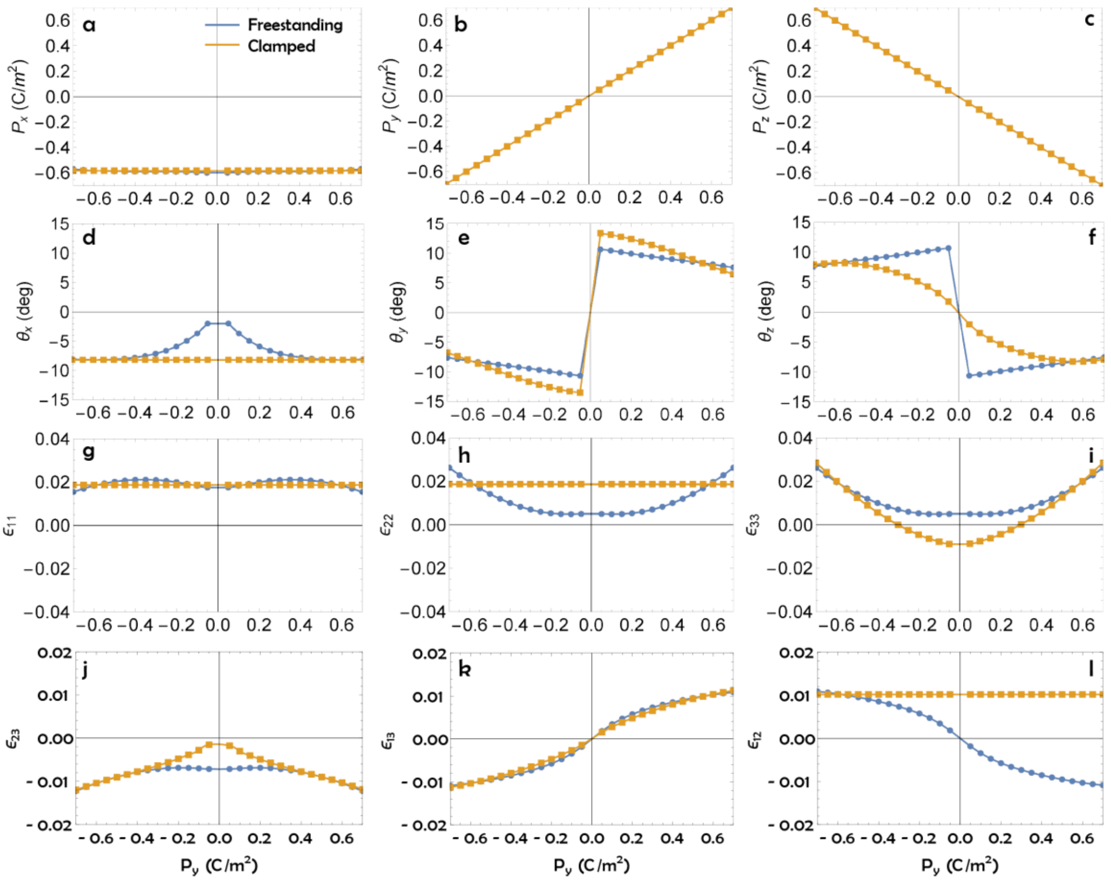

Figure S14 Extended Strong Strain + Tilt Clamping. Evolution of the polarization, octahedral tilts and strain tensor components during the 109° polarization switching. The curves denoted as "clamped" correspond to the "strong strain+tilt clamping" case (P_y=-P_z and θ_y=-θ_z are varied between their equilibrium values corresponding to up and down polarization directions while P_x, θ_x, as well as ϵ_{11}, ϵ_{22} and ϵ_{12} are fixed to their initial values. For each value of P_y and θ_y only ϵ_{33}, ϵ_{13} and ϵ_{23} components of the strain tensor are allowed to relax*).*

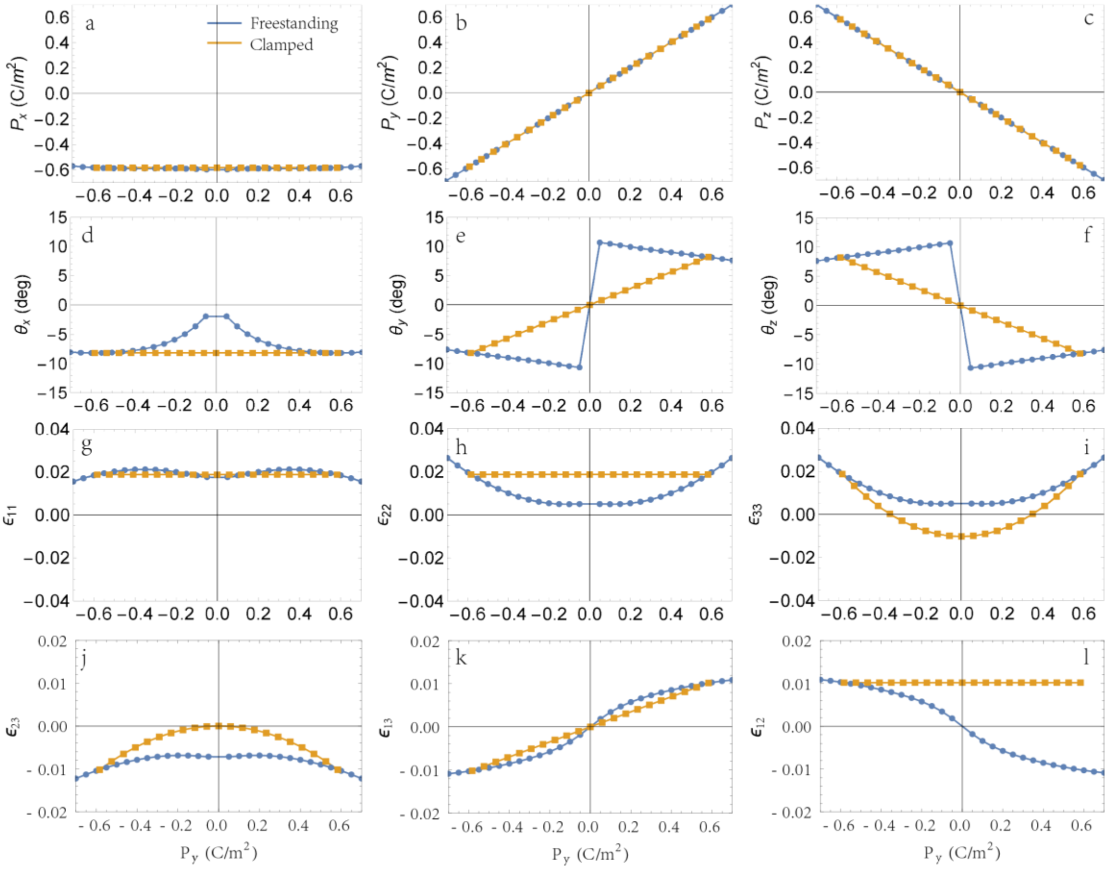

Figure S15 Ionic displacement patterns corresponding to (a) polar distortion (along *z* axis) and (b) FeO_6 octahedral rotations (around *z* axis). Arrows show only the directions of the ionic displacements and do not reflect their relative amplitudes. Bi cations are highlighted in blue, Fe cations in green and O anions in red.

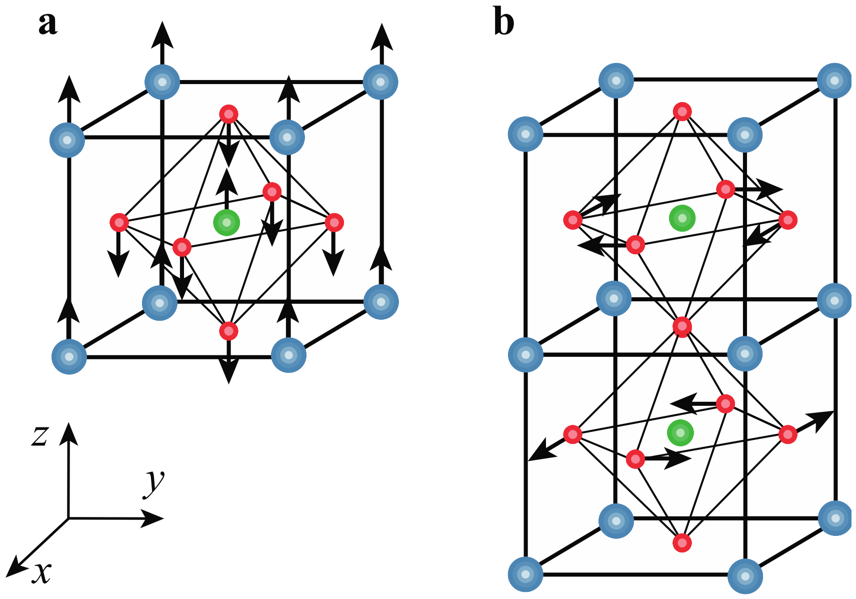

Table S1. "Static" switching voltage, $V_{c,0}$, and the frequency dispersion exponent, b, obtained from the fitting using the Ishibashi model[1]

**Sample**	$V_{c,0}$ **(V)**	b
BFO clamped film 100nm	3.12	0.25
BFO membrane 100nm	2.03	0.16
BFO clamped film 35nm	1.46	0.18
BFO membrane 35nm	0.81	0.15

Observed coercivity in ferroelectric hysteresis loop measurements is known to vary with the applied voltage frequency as $V_c = V_{c,0} + a\,f^b$,[7], [8] where $V_{c,0}$ is the thermodynamic limit of switching voltage (i.e., "Static" switching voltage, at zero frequency), f is the testing frequency, and a and b are obtained by fitting the frequency dependent data. The so-called "frequency dispersion" can be evaluated through the exponent b. As shown in **Fig. S7**, the data for all the samples fits well to this model, with fitting parameters shown (**Table S1**). In addition to the large reduction of static switching voltage estimated by the $V_{c,0}$, for each thickness when going from a clamped film to a membrane, it also interesting that the frequency dispersion of the freestanding membranes have both lower b values (indicating less dispersive behavior) and also less variability between thicknesses than their clamped film counterparts (clamped films vary from 0.25 at 100 nm to 0.18 at 25 nm, while the freestanding membranes vary only from 0.16 to 0.15 for the same thickness variation).

[1] F. Xue, Y. Gu, L. Liang, Y. Wang, and L.-Q. Chen, “Orientations of low-energy domain walls in perovskites with oxygen octahedral tilts,” *Phys. Rev. B*, vol. 90, no. 22, p. 220101, Dec. 2014, doi: 10.1103/PhysRevB.90.220101.

[2] G. Kresse and J. Furthmüller, “Efficient iterative schemes for ab initio total-energy calculations using a plane-wave basis set,” *Phys. Rev. B*, vol. 54, no. 16, pp. 11169–11186, Oct. 1996, doi: 10.1103/PhysRevB.54.11169.

[3] J. P. Perdew, K. Burke, and M. Ernzerhof, “Generalized Gradient Approximation Made Simple,” *Phys. Rev. Lett.*, vol. 77, no. 18, pp. 3865–3868, Oct. 1996, doi: 10.1103/PhysRevLett.77.3865.

[4] J. P. Perdew *et al.*, “Restoring the Density-Gradient Expansion for Exchange in Solids and Surfaces,” *Phys. Rev. Lett.*, vol. 100, no. 13, p. 136406, Apr. 2008, doi: 10.1103/PhysRevLett.100.136406.

[5] L. Q. Chen and J. Shen, “Applications of semi-implicit Fourier-spectral method to phase field equations,” *Comput. Phys. Commun.*, vol. 108, no. 2–3, pp. 147–158, Feb. 1998, doi: 10.1016/s0010-4655(97)00115-x.

[6] Y. L. Li, S. Y. Hu, Z. K. Liu, and L. Q. Chen, “Effect of substrate constraint on the stability and evolution of ferroelectric domain structures in thin films,” *Acta Mater.*, vol. 50, no. 2, pp. 395–411, Jan. 2002, doi: 10.1016/S1359-6454(01)00360-3.

[7] L. Liu, T. Rojac, D. Damjanovic, M. Di Michiel, and J. Daniels, “Frequency-dependent decoupling of domain-wall motion and lattice strain in bismuth ferrite,” *Nat. Commun.*, vol. 9, no. 1, p. 4928, Nov. 2018, doi: 10.1038/s41467-018-07363-y.

[8] Y. Ishibashi and H. Orihara, “A theory of D-E hysteresis loop,” *Integr. Ferroelectr.*, vol. 9, no. 1–3, pp. 57–61, Jun. 1995, doi: 10.1080/10584589508012906.
