# Supplementary material for: The role of lattice dynamics in ferroelectric switching
Source: Nat Commun. 2022 Mar 2;13:1110. doi: 10.1038/s41467-022-28622-z (PMC8891289; doi:10.1038/s41467-022-28622-z)
Supplement: Supplementary file 3 — Inventory of Supporting Information [file 41467_2022_28622_MOESM3_ESM.docx]

Inventory of Supporting Information:

1) Supplementary Information for the manuscript
